# Cellular mechanisms contributing to multiple stress tolerance in *Saccharomyces cerevisiae* strains with potential use in high-temperature ethanol fermentation

**DOI:** 10.1186/s13568-016-0285-x

**Published:** 2016-11-08

**Authors:** Yasin Kitichantaropas, Chuenchit Boonchird, Minetaka Sugiyama, Yoshinobu Kaneko, Satoshi Harashima, Choowong Auesukaree

**Affiliations:** 1 Department of Biology, Faculty of Science, Mahidol University, Rama VI Rd, Bangkok, 10400 Thailand; 2 Center of Excellence on Environmental Health and Toxicology, CHE, Ministry of Education, Bangkok, Thailand; 3 Department of Biotechnology, Faculty of Science, Mahidol University, Rama VI Rd, Bangkok, 10400 Thailand; 4 Department of Biotechnology, Graduate School of Engineering, Osaka University, 2-1 Yamadaoka, Suita, Osaka, 565-0871 Japan; 5 Department of Applied Microbial Technology, Faculty of Biotechnology and Life Science, Sojo University, 4-22-1 Ikeda, Kumamoto, 860-0082 Japan

**Keywords:** High-temperature ethanol fermentation, Multi-stress, Heat shock protein, Trehalose, Cell wall remodeling, Redox homeostasis, *Saccharomyces cerevisiae*

## Abstract

High-temperature ethanol fermentation has several benefits including a reduction in cooling cost, minimizing risk of bacterial contamination, and enabling simultaneous saccharification and fermentation. To achieve the efficient ethanol fermentation at high temperature, yeast strain that tolerates to not only high temperature but also the other stresses present during fermentation, e.g., ethanol, osmotic, and oxidative stresses, is indispensable. The C3253, C3751, and C4377 *Saccharomyces cerevisiae* strains, which have been previously isolated as thermotolerant yeasts, were found to be multiple stress-tolerant. In these strains, continuous expression of heat shock protein genes and intracellular trehalose accumulation were induced in response to stresses causing protein denaturation. Compared to the control strains, these multiple stress-tolerant strains displayed low intracellular reactive oxygen species levels and effective cell wall remodeling upon exposures to almost all stresses tested. In response to simultaneous multi-stress mimicking fermentation stress, cell wall remodeling and redox homeostasis seem to be the primary mechanisms required for protection against cell damage. Moreover, these strains showed better performances of ethanol production than the control strains at both optimal and high temperatures, suggesting their potential use in high-temperature ethanol fermentation.

## Introduction

The consumption of ethanol as an alternative fuel has been steadily rising. The budding yeast *Saccharomyces cerevisiae* is commonly used in industrial-scale ethanol production due to several advantages it offers, including a highly efficient ethanol fermentation ability and a relatively high tolerance to fermentation stress (Boulton and Quain 2006). Generally, the maximum ethanol yields of a conventional yeast fermentation process are attained at 25–30 °C, which is the optimal temperature for yeast growth (Hohmann and Mager 2003). However, ethanol fermentation at high temperature has a number of benefits such as reducing cooling costs, preventing bacterial contamination, and enabling simultaneous saccharification and fermentation (Abdel-Banat et al. 2010). During ethanol fermentation, yeast cells simultaneously encounter several stresses, e.g. osmotic, ethanol, and oxidative stresses (Gibson et al. 2007). To achieve the efficient ethanol fermentation at high temperature, yeast strain that tolerates to not only heat stress but also other stresses present during fermentation is therefore indispensable. Moreover, an understanding of cellular mechanisms required for protecting yeast cells from a wide range of physical and chemical stressors is essential for the improvement of multi-stress tolerance in yeast.

In our previous report, the continuous high-level expression of heat stress-responsive genes, including those encoding heat shock proteins (HSPs) and trehalose metabolic enzymes, was shown to be involved in an acquisition of thermotolerance in *S. cerevisiae* (Auesukaree et al. 2012). HSPs are known to function in folding unfolded and misfolded proteins, and mediating protein transport and degradation. In response to an accumulation of denatured proteins, the activation of heat shock transcription factor (HSF) is triggered, thereby leading to an upregulation of *HSP* genes expression (Verghese et al. 2012). In addition to heat stress, some stresses present during ethanol fermentation such as ethanol and oxidative stresses also induce protein denaturation (Cabiscol et al. 2000; Stanley et al. 2010). In agreement, the expression levels of *HSP* genes have been shown to increase when exposed to ethanol (Stanley et al. 2010), oxygen radicals (Gasch et al. 2000), and high sugar concentrations (Erasmus et al. 2003). In concert with HSPs, a disaccharide trehalose also plays an important role in preventing protein denaturation and aggregation through its protein binding activity to stabilize protein structure (Singer and Lindquist 1998). When exposed to protein-damaging stresses, the expression of trehalose metabolism-related genes such as *TPS1, TPS2,* and *NTH1* encoding trehalose-6-phosphate synthase/phosphatase, and neutral trehalase, respectively, is strikingly upregulated under the control of Msn2p and Msn4p transcription factors, thereby leading to an induction of trehalose synthesis (Parrou et al. 1997; Zähringer et al. 1997). The increase of intracellular trehalose levels have been observed under heat, osmotic, ethanol, and oxidative stress conditions (Benaroudj et al. 2001; Hounsa et al. 1998; Mahmud et al. 2010; Singer and Lindquist 1998). Moreover, the *TPS2* gene has been shown to be required for tolerance to several stresses present during fermentation, including ethanol, heat, osmotic, and oxidative stresses (Auesukaree et al. 2009). Based on these previous findings, it is therefore possible that cellular mechanisms responsible for thermotolerance may also play an important role in protecting yeast cells against other environmental stresses. Consistent with this idea, the thermotolerant *S. cerevisiae* KNU5377 strain has been reported to be also tolerant to ethanol, and oxidative stresses (Kim et al. 2013).

Although oxygen (O_2_) is necessary for promoting yeast growth at early stages of fermentation and for maintaining yeast at optimum condition for effective fermentation, incomplete oxygen metabolism potentially causes a generation of reactive oxygen species (ROS), derivative forms of O_2_, via the mitochondrial electron transport chain (Gibson et al. 2007). The major species of ROS produced by the cells are superoxide anion, hydrogen peroxide, and hydroxyl radical. Imbalance of intracellular ROS levels results in lipid peroxidation, protein carbonylation, and nucleic acid damage (Herrero et al. 2008). The ROS accumulation and oxidative damage to cell structures were observed in *S. cerevisiae* wine strains during fermentation (Landolfo et al. 2008). To protect cells from oxidative damage, the endogenous antioxidant defense systems, such as superoxide dismutase, catalase, and glutathione, play a crucial role in ROS scavenging (Herrero et al. 2008). Among these, cytosolic Cu/Zn superoxide dismutase Sod1p has been shown to be required for tolerance to not only oxidative stress but also heat, ethanol, and osmotic stresses (Auesukaree et al. 2009). It is therefore likely that an ability to control redox homeostasis is essential for maintenance of yeast metabolisms during fermentation.

Cell wall is the rigid outermost layer of yeast cells, which is important for supporting cell structures and for protecting the cell from chemical and physical damages. The yeast cell wall is composed of chitins, glucans, mannans, and glycoproteins (Klis et al. 2006). When the cell wall is disturbed, cell wall stress signals are transmitted through the cell wall integrity (CWI) pathway to activate the expression of a set of genes involved in cell wall biogenesis, thereby resulting in a strengthened cell wall (Levin 2011). Since the cell wall is the first line of defense against external stresses, the strength of cell wall and the effective cell wall remodeling may be essential for tolerance to various environmental stresses.

During fermentation, yeast cells continuously encounter osmotic stress due to high sugar concentrations (Gibson et al. 2007). To cope with the high osmolarity, a production of intracellular osmolyte glycerol is rapidly induced upon osmotic shock via enhanced expression of *GPD1* and *GPD2* genes encoding isoenzymes of NAD-dependent glycerol 3-phosphate dehydrogenase under the regulation of high osmolarity glycerol (HOG) pathway (Ansell et al. 1997). Furthermore, glycerol has been shown to be involved in protecting cells against high temperature and oxidative stress (Pahlman et al. 2001; Siderius et al. 2000), possibly through its role in reoxidizing excess NADH (Ansell et al. 1997).

The main objectives of this study were to screen for yeast strains displaying tolerance to multiple stresses present during high-temperature ethanol fermentation, i.e., heat, ethanol, osmotic, oxidative, and cell wall stresses, and to investigate cellular mechanisms responsible for multiple stress tolerance in these strains. The role of HSP, trehalose, glycerol, redox balance, and cell wall remodeling in tolerance to these environmental stresses were determined. In addition, performances of high-temperature ethanol fermentation by these multiple stress-tolerant strains were also evaluated.

## Materials and methods

### Strains and media

The *S*. *cerevisiae* strains used in this study were laboratory strain W303 (*MATa/MATa ade2*-*1/ade2*-*1 his3*-*11,15/his3*-*11,15 leu2*-*3,112/leu2*-*3,112 trp1*-*1/trp1*-*1 ura3*-*1/ura3*-*1*) (Kurtzman and Robnett 1998), industrial ethanol-producing strain TISTR5606 obtained from the Thailand Institute of Scientific and Technological Research, and the thermotolerant strains C3225, C3253, C3723, C3751, C3867, C3891, C4275, and C4377 (Auesukaree et al. 2012) obtained from the yeast collection of the Department of Biotechnology, Faculty of Science, Mahidol University. YPDA (1% yeast extract, 2% peptone, 2% glucose, and 0.04% adenine) and YPDA10 (YPDA medium containing 10% glucose) media were prepared as described previously (Benjaphokee et al. 2012; Burke et al. 2000).

### Spot susceptibility assay

Yeast cells precultivated to log-phase (OD_600_ = 0.8–1) in YPDA media were harvested, resuspended in sterile water to an OD_600_ of 1.0, and then serially tenfold diluted. Aliquots of 3 µL were spotted onto YPDA agar plates containing 17% ethanol, 18% glucose, 18% sorbitol, 8 mM H_2_O_2_, or 100 mg L^−1^ calcofluor white (CFW), and incubated at 30 °C for 3 days. To examine the growth at high temperature, the plates were incubated at 40 °C.

### Growth analysis in liquid media

Log-phase cells precultivated in YPDA media were harvested, and transferred into YPDA media containing 5% ethanol, 30% glucose, 30% sorbitol, 1.5 mM H_2_O_2_, or 50 mg L^−1^ CFW to a starting OD_600_ of 0.1. The cultures were incubated with shaking (200 rpm) at 30 °C. To determine the growth at high temperature, the cultures were incubated at 40 °C. Cell growth was monitored at 2-h intervals for 24 h by measuring the OD_600_. Specific growth rates (*μ*) were calculated from the growth curves.

### RNA isolation and quantitative RT-PCR assay

Total RNA from log-phase cells was isolated by using a FavorPrep Tissue Total RNA Purification Mini Kit (Favorgen, Ping-Tung, Taiwan) following manufacturer’s instructions. Each RNA sample was converted to cDNA by using iScript™ cDNA synthesis kit with reverse transcriptase (Bio-Rad, Hercules, CA, USA). Quantitative RT-PCR experiments were performed on a LightCycler Real-Time PCR (Roche, Mannheim, Germany) using KAPA SYBR FAST qPCR kit (Kapa Biosystems, Wilmington, MA, USA) and 200 nM specific primer pairs (SSA4-1088F and SSA4-1150R for *SSA4* gene, HSP82-387F and HSP82-465R for *HSP82* gene, and ACT1-458F and ACT1-529R for *ACT1* gene) as described previously (Auesukaree et al. 2012). Relative gene expression was calculated using the 2^−*ΔΔCT*^ method and normalized to *ACT1* mRNA levels.

### Trehalose measurement

The intracellular trehalose content was measured using the modified Anthrone method as described previously (Benjaphokee et al. 2012). Briefly, log-phase cells were suspended in 400 µL of 0.5 M trichloroacetic acid (TCA), and mixed vigorously at room temperature for 40 min. The crude extract was collected by centrifugation at 18,000×*g* for 2 min. Aliquots of 200 µL were mixed with 1 mL of cold Anthrone reagent and incubated at 100 °C for 10 min prior to the measurement of A_620_.

### Measurement of intracellular ROS

The intracellular ROS levels were determined by using the oxidant-sensitive probe 2′,7′-dichlorodihydrofluorescein diacetate (DCFH-DA; Sigma, St. Louis, MO, USA) as described previously (Wu et al. 2007). Briefly, log-phase cells were treated with 10 mM DCFH-DA in culture media for 1 h. Cells were then harvested, resuspended in PBS (phosphate-buffered saline), and disrupted with glass beads. The supernatants collected by centrifugation were used for the measurement of fluorescence intensities at an excitation wavelength of 490 nm and an emission wavelength of 524 nm by the SpectraMax M3 microplate reader (Molecular Devices, Sunnyvale, CA, USA). The florescence intensity values were normalized to protein levels in the supernatants.

### Zymolyase susceptibility test

Susceptibility to Zymolyase was examined as described previously (Charoenbhakdi et al. 2016). Briefly, log-phase cells were diluted to an OD_600_ of 0.5 in TE buffer [10 mM Tris–HCl and 1 mM EDTA (pH 7.5)] containing 100 mg L^−1^ (1 U) Zymolyase 20T (Zymo Research, Orange, CA, USA). The OD_600_ was measured at 15-min intervals for 2 h by the Wallace Victor 1420 microplate reader (PerkinElmer, Waltham, MA, USA).

### Measurement of intracellular glycerol

The intracellular glycerol levels were measured by using the Free Glycerol Reagent kit (Sigma-Aldrich, St. Louis, MO, USA) following manufacturer’s instructions. Log-phase cells were harvested, resuspended in sterile water, and lysed by heating at 95 °C for 10 min. Five microlitre of supernatants obtained by centrifugation at 13,000×*g* for 3 min were mixed with 800 µL of free glycerol reagent, and then incubated at 30 °C for 15 min prior to the measurement of absorbance at 540 nm.

### Ethanol fermentation

Yeast cells precultivated to log-phase (OD_600_ = 0.8–1) in YPDA media were inoculated into 125-mL Erlenmeyer flask containing 50 mL of YPDA10 medium. The initial cell density was adjusted to OD_600_ of 0.1. The flasks were sealed with parafilm to allow fermentation to be carried out under semi-anaerobic conditions, and incubated in an orbital shaker at 150 rpm at 30 or 40 °C for 48 h. At the indicated times, the fermentation samples were collected and centrifuged at 13,000×*g* for 3 min. Ethanol and glucose concentrations were determined by using the ethanol assay F-kit (Roche Diagnostics, Basel, Switzerland) and the glucose assay F-kit (Roche Diagnostics, Basel, Switzerland), respectively, following manufacturer’s instructions.

### Data analysis

All experiments were independently performed at least three times and expressed as means with standard deviations. Analysis of variance was conducted by one-way analysis of variance (ANOVA) using least significant difference method (LSD) on the SPSS statistical package (version 18.0 for Windows, SPSS Inc., Chicago, IL, USA). The level of statistical significance was set at *p* < 0.05.

## Results

### Screening for yeast strains displaying multiple stress tolerance

**Fig. 1:**
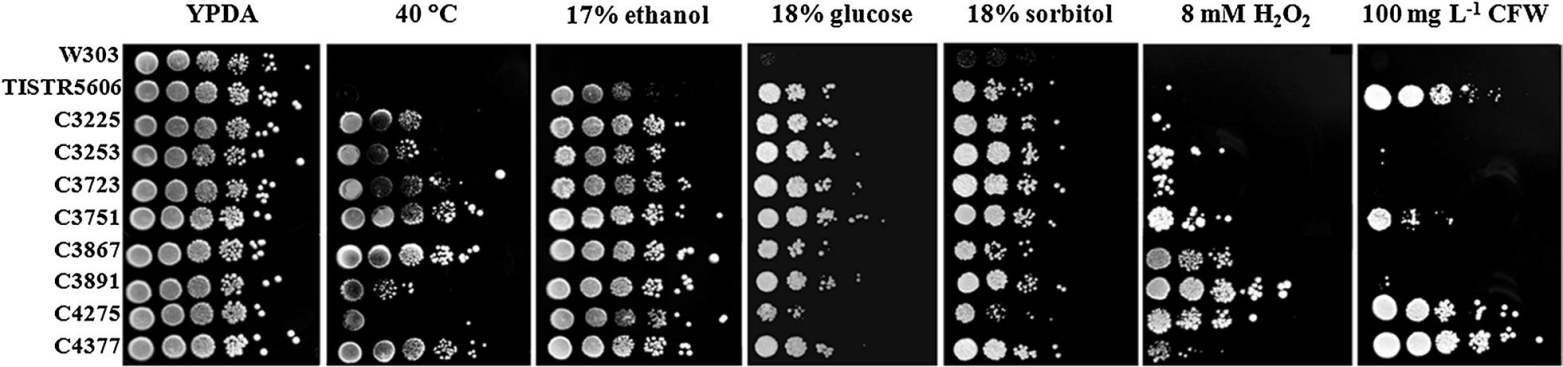
Growth of the multiple stress-tolerant *S. cerevisiae* strains on YPDA agar plates containing 17% (v/v) ethanol, 18% (w/v) glucose, 18% (w/v) sorbitol, 8 mM H_2_O_2_, and 100 mg L^−1^ CFW at 30 °C. For heat stress, YPDA agar plates were incubated at 40 °C

Since several cellular mechanisms responsible for thermotolerance, such as high levels of HSPs and trehalose, have been suggested to be important for the protection of yeast cells against various environmental stresses, the thermotolerant yeasts may exhibit cross-resistance to other stresses, especially those present during fermentation. We first confirmed the thermotolerant phenotype of our natural *S*. *cerevisiae* isolates, which have been isolated from Thai fruits based on their thermotolerant characteristics (Auesukaree et al. 2012), by examining the growth at 40 °C using serial-dilution spot test. Four of our yeast strains (C3723, C3751, C3867, and C4377) were highly tolerant to high temperature, whereas the C3225 and C3253 strains were moderately thermotolerant (Fig. 1). During fermentation, yeast cells are exposed to several environmental stresses including ethanol, osmotic, cell wall, and oxidative stresses, the ability of these strains to tolerate these stresses was next investigated by examining the growth on YPDA agar plates containing 17% (v/v) ethanol, 18% (w/v) glucose, 18% (w/v) sorbitol, 100 mg L^−1^ cell wall stress-inducing agent calcofluor white (CFW), or 8 mM H_2_O_2_. Among these strains, the C4377 and C3751 strains were tolerant to all stresses examined, whereas the C3253 was tolerant to three stresses, i.e., ethanol, osmotic, and oxidative stresses (Fig. 1). These results suggest that thermotolerant yeasts have ability to tolerate multiple stresses.

To quantitate the growth of these strains under stress conditions, we measured the specific growth rates of cells grown in YPDA media containing 5% (v/v) ethanol, 30% (w/v) glucose, 30% (w/v) sorbitol, 50 mg L^−1^ CFW, or 1.5 mM H_2_O_2_. For heat stress, the experiments were performed at 40°C. Under heat stress condition, the C3751 and C4377 strains showed the highest specific growth rate, while the C3225, C3253, and C3723 strains exhibited significant higher specific growth rates than both control strains W303 and TISTR5606 (Table 1). At 40 °C, although the specific growth rates of C3867, C3891, and C4275 strains were similar to that of the TISTR5606 strain, their growth rates were significantly higher than that of the W303 strain (Table 1). When grown under other stress conditions, the C4377 strain exhibited the highest specific growth rates under ethanol, high glucose, and cell wall stress conditions, and its growth rate under high sorbitol condition was higher than the control strains (Table 1). Whereas the C3253 and C3751 strains showed higher specific growth rates than the control strains under three stress conditions (i.e. the C3253 strain grown under high glucose, oxidative, and cell wall stress conditions; and the C3751 strain grown under ethanol, high glucose, and oxidative stress conditions). On the other hand, the growth rates of the other thermotolerant strains under these stress conditions were similar or lower than those of the industrial ethanol-producing TISTR5606 strain (Table 1). Therefore, the multiple stress-tolerant C4377, C3751, and C3253 strains were then used in the next investigations.

**Table 1:** Growth rates of the multiple stress-tolerant *S. cerevisiae* strains under stress conditions

Strain	Specific growth rate (μ, h^−1^)						
	YPDA	40 °C	5% ethanol	30% glucose	30% sorbitol	1.5 mM H_2_O_2_	50 mg L^−1^ CFW
W303	0.24 ± 0.09	0.03 ± 0.01	0.03 ± 0.01	0.04 ± 0.02	0.04 ± 0.01	0.06 ± 0.04	0.02 ± 0.01
TISTR5606	0.69 ± 0.08	0.22 ± 0.11	0.19 ± 0.03	0.17 ± 0.03	0.20 ± 0.03	0.48 ± 0.10	0.09 ± 0.01
C3225	0.61 ± 0.03	0.48 ± 0.02	0.07 ± 0.03	0.18 ± 0.02	0.23 ± 0.05	0.42 ± 0.06	0.04 ± 0.01
C3253	1.15 ± 0.12^a^	0.60 ± 0.03	0.16 ± 0.02	0.27 ± 0.02	0.26 ± 0.07	0.72 ± 0.12^a^	0.14 ± 0.01^a^
C3723	0.69 ± 0.03	0.43 ± 0.13	0.15 ± 0.04	0.23 ± 0.04	0.25 ± 0.05	0.47 ± 0.06	0.04 ± 0.01
C3751	0.99 ± 0.03	0.88 ± 0.12^a^	0.23 ± 0.03	0.35 ± 0.05^a^	0.22 ± 0.04	0.69 ± 0.07^a^	0.06 ± 0.01
C3867	0.80 ± 0.03	0.35 ± 0.02	0.12 ± 0.01	0.23 ± 0.03	0.22 ± 0.02	0.52 ± 0.08	0.05 ± 0.02
C3891	1.15 ± 0.16^a^	0.38 ± 0.08	0.12 ± 0.03	0.27 ± 0.06	0.22 ± 0.04	0.80 ± 0.06^a^	0.05 ± 0.02
C4275	0.63 ± 0.10	0.25 ± 0.02	0.19 ± 0.04	0.13 ± 0.04	0.14 ± 0.01	0.30 ± 0.08	0.11 ± 0.02
C4377	1.00 ± 0.07	0.91 ± 0.05^a^	0.32 ± 0.04^a^	0.28 ± 0.05	0.34 ± 0.04^a^	0.28 ± 0.06	0.15 ± 0.02^a^

^a^Indicate the highest specific growth rate of each growth condition

### Continuous expression of HSP genes in response to environmental stresses

**Fig. 2:**
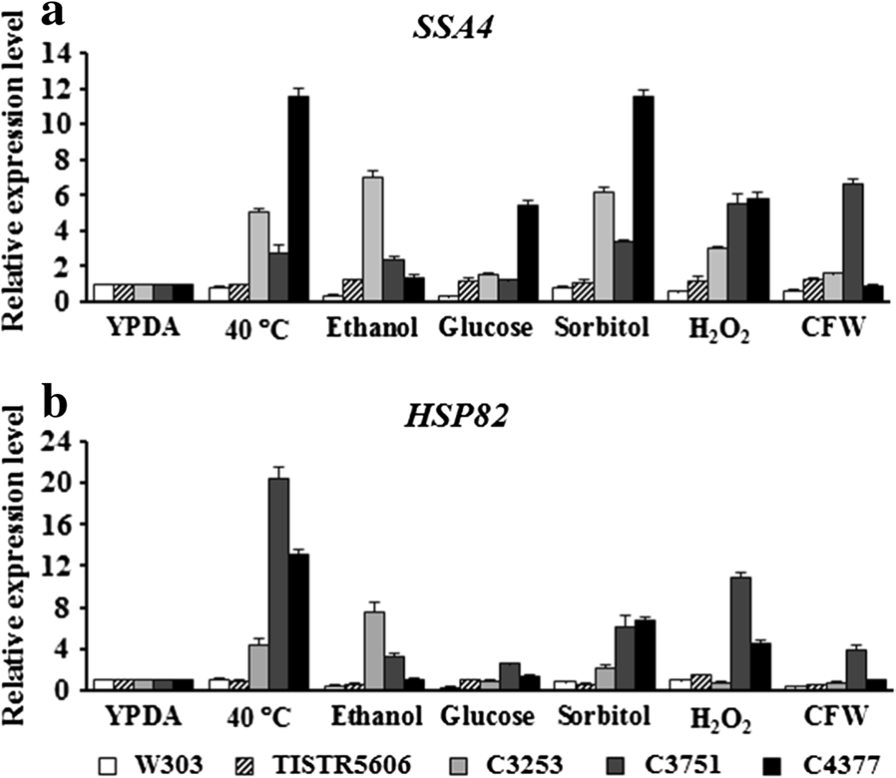
Expression levels of *SSA4* (**a**) and *HSP82* genes (**b**) of the multiple stress-tolerant *S. cerevisiae* strains after exposure to high temperature (40 °C), 17% (v/v) ethanol, 30% (w/v) glucose, 30% (w/v) sorbitol, 8 mM H_2_O_2_, and 100 mg L^−1^ CFW for 12 h

Generally, in response to an accumulation of denatured proteins, the expression of *HSP* genes is transiently upregulated in order to refold misfolded proteins and to prevent protein aggregation (Verghese et al. 2012). However, once the intracellular HSPs reach the sufficient protection levels, the expression is then repressed (Auesukaree et al. 2012; Verghese et al. 2012). Contrary to this prevailing model, we have previously shown that, in the thermotolerant *S. cerevisiae* C3723 and C3867 strains, the expression of *SSA4* and *HSP82* genes encoding heat shock proteins of HSP70 and HSP90 family, respectively, were maintained at high levels even after long-term exposure to heat stress, suggesting that the continuous expression of these *HSP* genes may contribute to thermotolerance (Auesukaree et al. 2012). To examine whether the continuous expression of *HSP* genes is the common characteristic of thermotolerant yeasts, the expression levels of *SSA4* and *HSP82* genes in the C3253, C3751, and C4377 strains grown at 40 °C for 12 h were determined. Consistent with the previous results in the C3723 and C3867 strains (Auesukaree et al. 2012), the expression levels of both genes in the C3253, C3751, and C4377 strains were significantly higher than those in the control strains (Fig. 2a, b), suggesting that the continuous expression of *HSP* genes may be involved in an acquisition of thermotolerant phenotype.

To further examine the role of continuous expression of *HSP* genes in tolerance to other stresses, the expression levels of *SSA4* and *HSP82* genes in the C3253, C3751, and C4377 strains were determined after exposed to 17% (v/v) ethanol, 30% (w/v) glucose, 30% (w/v) sorbitol, 8 mM H_2_O_2_, and 100 mg L^−1^ CFW. In the case of C3751 strain, the expression levels of both genes were maintained at high levels under almost all stress conditions tested (except for *SSA4* expression under high glucose condition) (Fig. 2a, b). These results suggest that the continuous expression of *HSP* genes may be one of those molecular mechanisms important for multiple stress tolerance in the C3751 strain. For the C3253, and C4377 strains, the high expression of both genes were observed in the C3253 strain treated with ethanol and sorbitol, and the C4377 strain treated with sorbitol and H_2_O_2_ (Fig. 2a, b). These findings suggest that the continuous expression of *HSP* genes is required for protecting the C3253 and C4377 cells against some stresses that may cause severe damage to their proteins.

### Trehalose accumulation upon exposure to environmental stresses

**Fig. 3:**
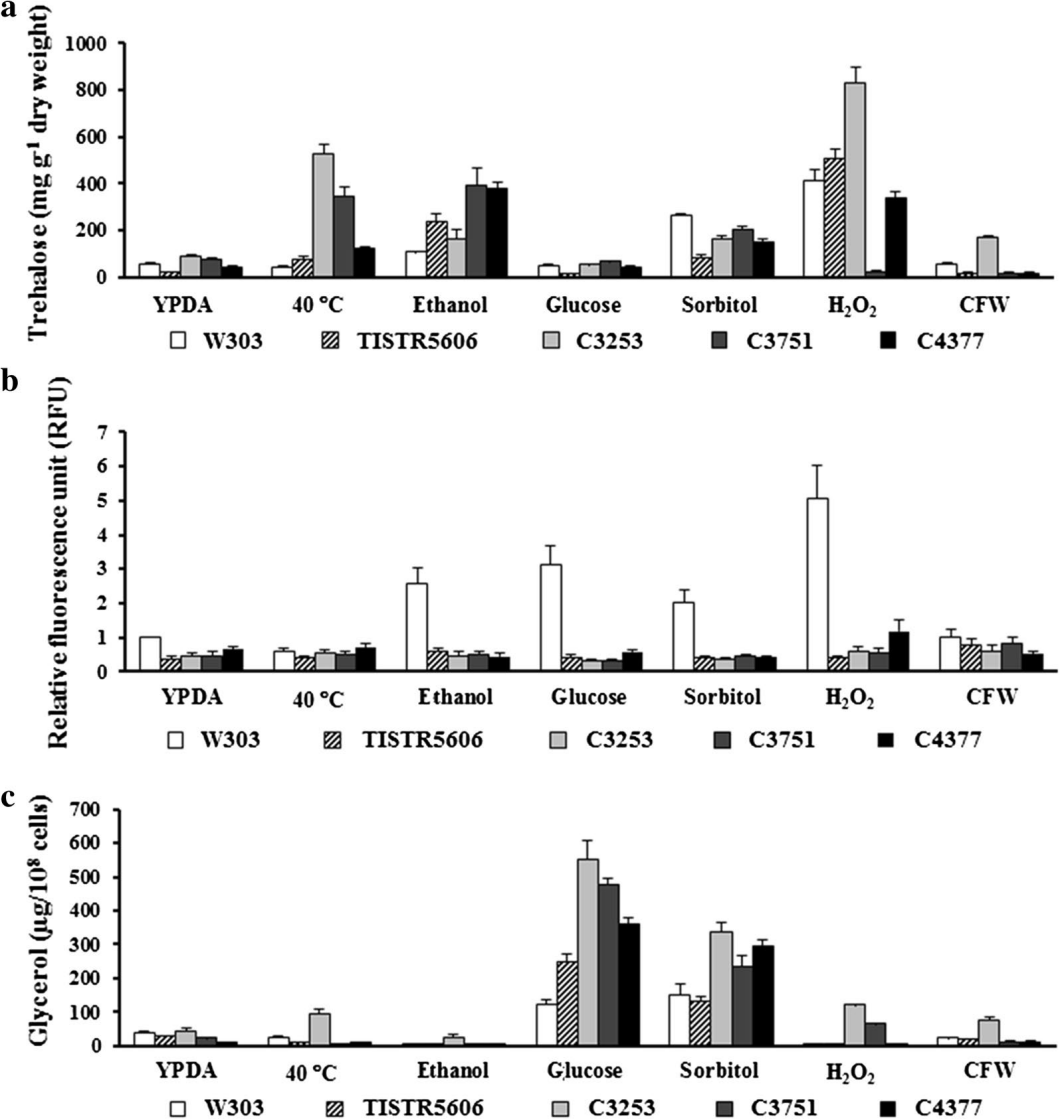
**a** Trehalose accumulations, **b** intracellular ROS levels, and **c** glycerol accumulations of the multiple stress-tolerant *S. cerevisiae* strains after exposure to high temperature (40 °C), 17% (v/v) ethanol, 30% (w/v) glucose, 30% (w/v) sorbitol, 8 mM H_2_O_2_, and 100 mg L^−1^ CFW for 4 h

In addition to HSPs, trehalose is also involved in stabilizing protein structures in order to prevent protein denaturation and aggregation (Singer and Lindquist 1998). The expression of genes involved in trehalose metabolism is rapidly upregulated in response to protein-denaturing stress such as heat stress, leading to the increased intracellular trehalose levels (Benaroudj et al. 2001; Hounsa et al. 1998; Mahmud et al. 2010; Singer and Lindquist 1998). Moreover, we have previously shown that *TPS2* gene encoding trehalose-6-phosphate phosphatase is required for tolerance to heat, ethanol, oxidative, and osmotic stresses (Auesukaree et al. 2009). It is therefore likely that the intracellular trehalose accumulation may be important for multiple stress tolerance in our strains. To test this hypothesis, we measured the intracellular trehalose levels in these strains after exposing them to high temperature, ethanol, glucose, sorbitol, H_2_O_2_, and CFW. The trehalose levels in almost all strains significantly increased upon exposure to high temperature, ethanol, sorbitol, and H_2_O_2_ (Fig. 3a). In the cases of heat and ethanol stresses, the strains exhibiting high growth rates under these stress conditions were found to accumulate high levels of intracellular trehalose (Fig. 3a; Table 1). These results suggest that the level of trehalose accumulation may be another factor important for tolerance to several environmental stresses, particularly protein damage-inducing stresses.

### The ability to maintain intracellular redox homeostasis is important for tolerance to environmental stresses

Although the conventional ethanol fermentation is carried out under semi-anaerobic conditions, the ROS accumulation and oxidative damage to cell structures were observed in the *S. cerevisiae* wine strains during fermentation in high-sugar-containing media (Landolfo et al. 2008). In addition, *SOD1* gene encoding Cu/Zn-superoxide dismutase, an important antioxidant enzyme, was required for tolerance to several stresses occurring during fermentation, including heat, ethanol, osmotic, and oxidative stresses (Auesukaree et al. 2009). Taken together, the ability to maintain intracellular redox balance may be necessary for yeast growth upon exposure to multiple stresses present during fermentation. To test this possibility, we measured the intracellular ROS levels after grown in the presence of ethanol, glucose, sorbitol, H_2_O_2_, and CFW at 30 °C, or at 40 °C for heat stress. In the W303 strain, the intracellular ROS levels were dramatically increased after being challenged with not only the oxidant H_2_O_2_ but also ethanol, glucose, and sorbitol (Fig. 3b), suggesting the effects of ethanol and hyperosmolarity on inducing endogenous oxidative stress. On the other hand, the intracellular ROS levels in the other strains, which exhibited higher growth rates than the W303 under these stress conditions, were not significantly increased after treatments (Fig. 3b). These results suggest that these multiple stress-tolerant strains may have the ability to minimize the intracellular ROS levels.

### Cell wall remodeling in response to environmental stresses

**Fig. 4:**
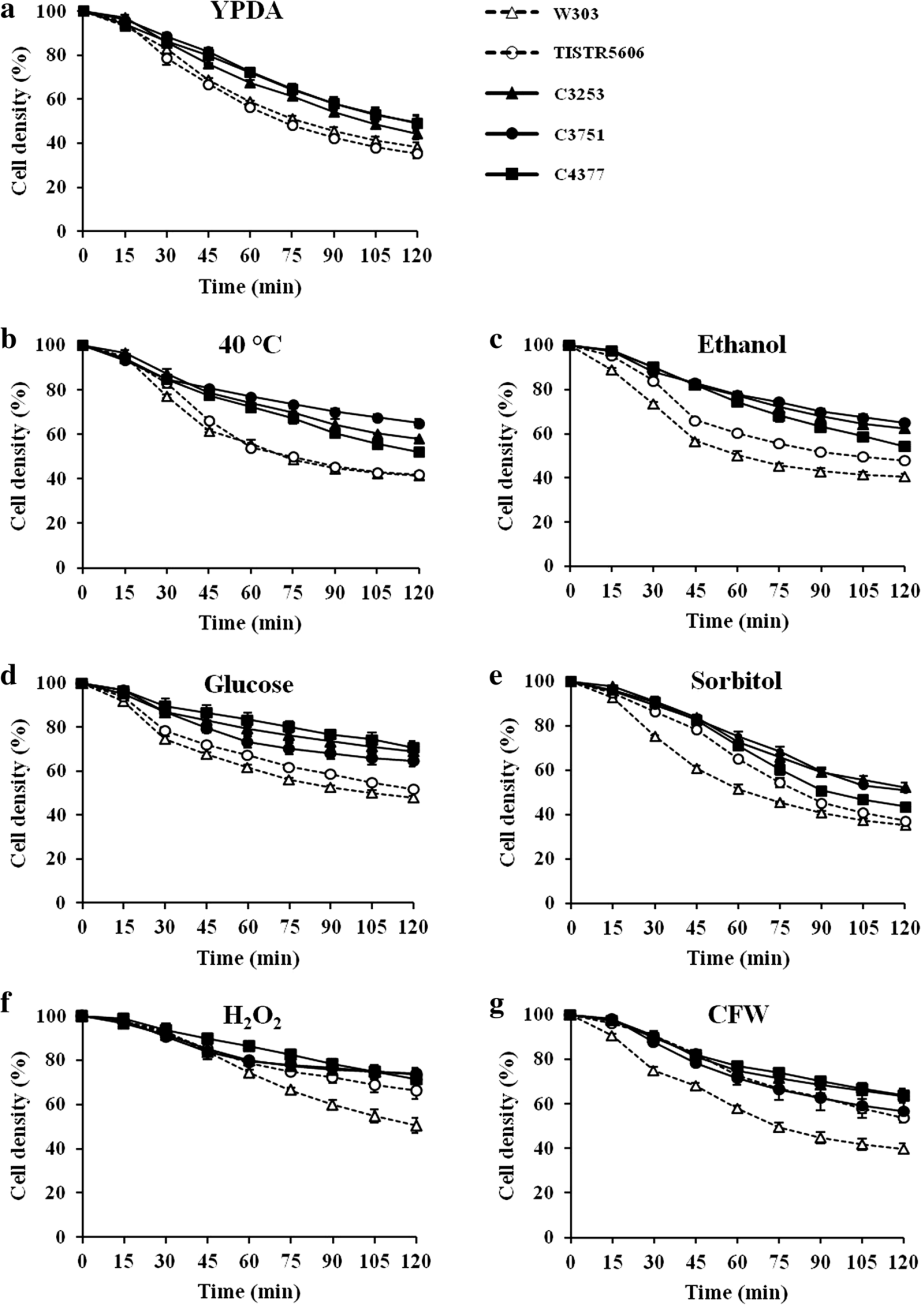
**a** Susceptibility to Zymolyase of the multiple stress-tolerant *S. cerevisiae* strains, after exposure to high temperature (40 °C) (**b**), 17% (v/v) ethanol (**c**), 30% (w/v) glucose (**d**), 30% (w/v) sorbitol (**e**), 8 mM H_2_O_2_ (**f**), and 100 mg L^−1^ CFW (**g**) for 4 h

The cell wall is the first line of defense of yeast cells against external stresses (Klis et al. 2006). It is known that, in response to cell wall-disturbing stresses, the architecture of cell wall is remodeled through the activation of the CWI signaling pathway, leading to a more robust wall (Levin 2011). To monitor the cell wall remodeling in response to these environmental stresses, the susceptibilities of these strains to Zymolyase, the cell wall-degrading enzyme whose major activities are β-1,3-glucanase and β-1,3-glucan laminaripentaohydrolase, were determined after stress challenges. In addition to the cell wall-disturbing agent CFW, the increased resistances to Zymolyase were also observed after exposures to high temperature, ethanol, high glucose, and H_2_O_2_ (Fig. 4a–g), suggesting that the cell wall remodeling is induced in response to cell wall damages caused by heat, ethanol, osmotic, and oxidative stresses. Furthermore, it should be noted that the multiple stress-tolerant strains were more resistant to Zymolyase than the control strains under both stress and non-stress conditions (Fig. 4a–g). These findings suggest that the cell walls of these multiple stress-tolerant strains are natively more robust than those of the control strains and that these strains have a high ability to induce effective cell wall remodeling to protect themselves against external stresses.

### Glycerol accumulation upon exposure to osmotic stress

During yeast osmoadaptation against high sugar concentrations in fermentation, the biosynthesis of glycerol is promoted in order to balance osmotic pressure across the yeast plasma membrane (Gibson et al. 2007; Hohmann 2002). In addition, glycerol is also involved in protecting yeast cells against high temperature and oxidative stress (Pahlman et al. 2001; Siderius et al. 2000). To examine the abilities of these multiple stress-tolerant strains in accumulating glycerol in response to various environmental stresses, the intracellular glycerol contents in these strains were measured after stress exposures. The intracellular glycerol contents in all yeast strains tested were greatly increased only after treatments with high concentrations of glucose and sorbitol, which are known to induce osmotic stress (Fig. 3c). Moreover, under these osmotic stress conditions, the multiple stress-tolerant strains accumulated higher levels of intracellular glycerol than the control strains (Fig. 3c), suggesting the important role of glycerol in conferring enhanced osmotolerance to these strains. It should be noted that, in the C3253 strain, the glycerol production was slightly induced after exposures to heat, oxidative, and cell wall stresses (Fig. 3c), raising the possibility that glycerol may also be important for protecting the C3253 strain against other environmental stresses.

### Cellular mechanisms important for tolerance to simultaneous multi-stress

During fermentation, yeast cells are simultaneously exposed to a variety of stresses (Gibson et al. 2007). The abilities of these multiple stress-tolerant strains to tolerate simultaneous multi-stress mimicking fermentation stress were then determined by examining the growth of C3253, C3751, and C4377 strains in the presence of 5% (v/v) ethanol and 10% (w/v) glucose at 40 °C. Based on the results of serial-dilution spot test, the growth of all multiple stress-tolerant strains was better than the W303 strain but apparently similar to that of the TISTR5606 strain (data not shown). However, when measuring their specific growth rates in liquid media, these multiple stress-tolerant strains showed higher growth rates than both control strains (W303 and TISTR5606) (Table 2), indicating that these strains are resistant to simultaneous multi-stress.

**Table 2:** Growth rates of the multiple stress-tolerant *S. cerevisiae* strains under multi-stress condition

Strain	Specific growth rate (μ, h^−1^)	
	YPDA	Multi-stress
W303	0.24 ± 0.09	0.02 ± 0.01
TISTR5606	0.69 ± 0.08	0.09 ± 0.03
C3253	1.15 ± 0.12	0.23 ± 0.02
C3751	0.99 ± 0.03	0.16 ± 0.06
C4377	1.00 ± 0.07	0.20 ± 0.01

**Fig. 5:**
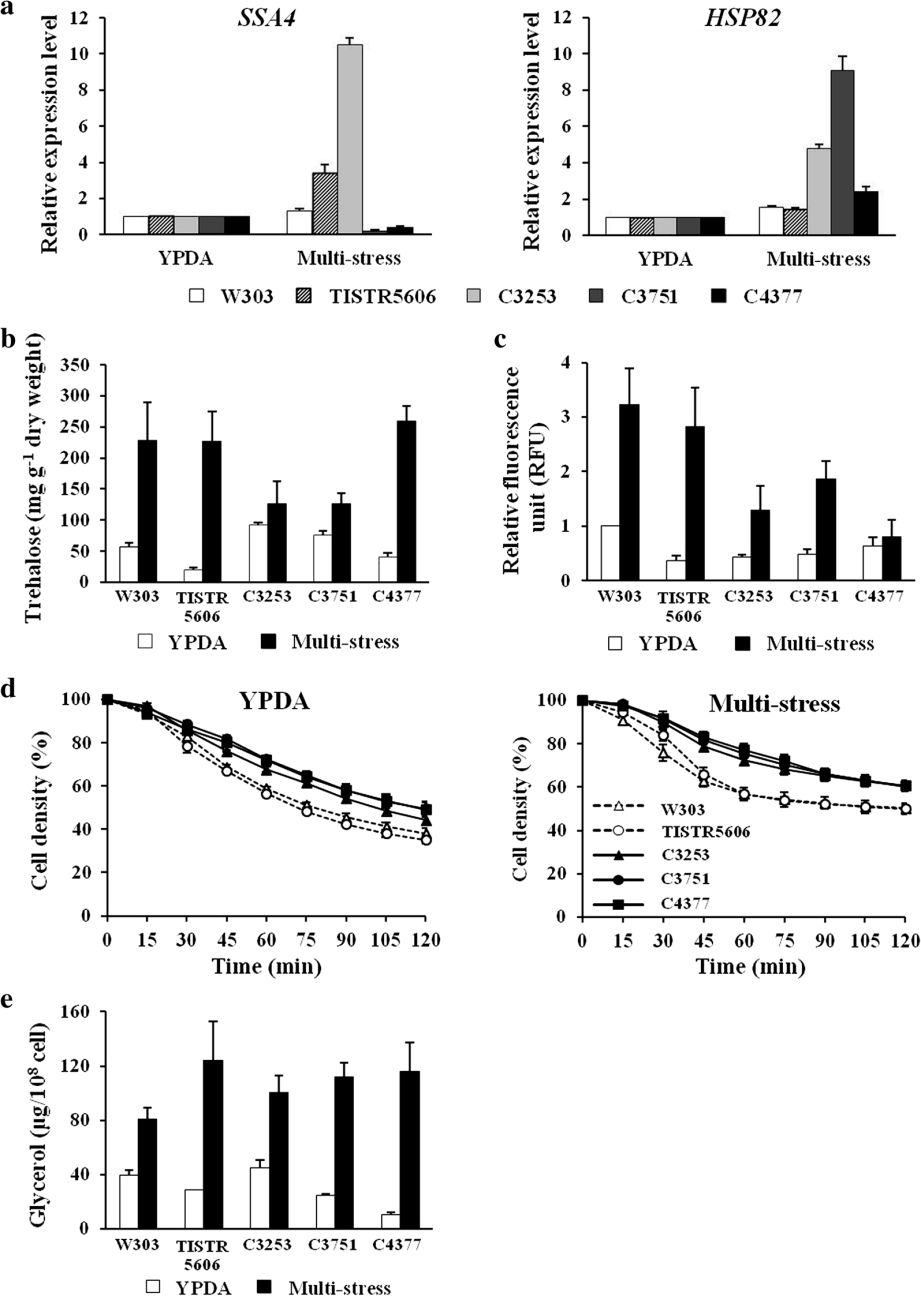
Cellular responses to simultaneous multi-stress. **a** *SSA4* and *HSP82* mRNA levels, **b** trehalose accumulations, **c** ROS levels, **d** susceptibility to Zymolyase, and **e** glycerol accumulations of the multiple stress-tolerant *S. cerevisiae* strains after exposure to simultaneous multi-stress (5% (v/v) ethanol, 10% (w/v) glucose, and incubation temperature of 40 °C)

To investigate cellular mechanisms involved in response to simultaneous multi-stress, *HSP* genes expression, trehalose accumulation, intracellular ROS level, cell wall remodeling, and glycerol accumulation in these tolerant strains were determined after being exposed to simultaneous multi-stress (5% (v/v) ethanol, 10% (w/v) glucose, and an incubation temperature of 40 °C). In response to simultaneous multi-stress, the remodeled cell walls (increased resistances to Zymolyase) and the low intracellular ROS levels were observed in the multiple stress-tolerant strains (Fig. 5c, d). In contrast to the cases of single-stress treatments (Figs. 2, 3, 4) the continuous expression of *HSP* genes, i.e. *SSA4* and *HSP82*, upon exposure to simultaneous multi-stress was found only in the C3253 strain, while the C3751 strain exhibited the continuous expression of only the *HSP82* gene (Fig. 5a). In addition, even after multi-stress treatment, the intercellular glycerol and trehalose contents of these multiple stress-tolerant strains were similar or lower than those of the control strains (Fig. 5b, e). Based on these observations, it is likely that, in our multiple stress-tolerant strains, the cell wall remodeling and the maintenance of redox balance are the primary mechanisms that respond to simultaneous multi-stress.

### Performance of high-temperature ethanol fermentation by thermotolerant yeast strains

**Fig. 6:**
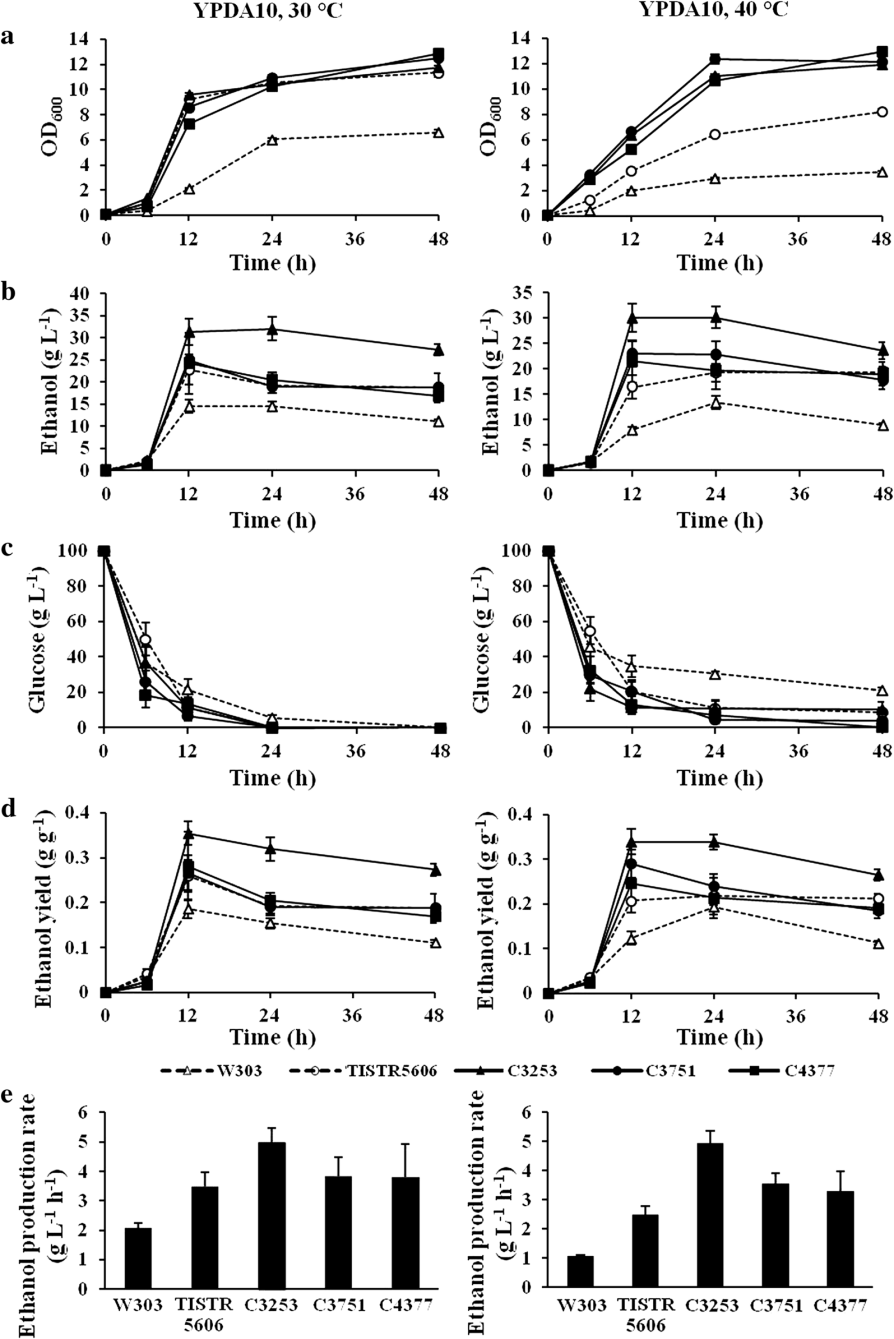
Ethanol fermentation performances of the multiple stress-tolerant *S. cerevisiae* strains in YPDA10 media at 30 (**a**, **c**, **e**, **g**, and **i**) and 40 °C (**b**, **d**, **f**, **h**, and **j**). The growth (**a** and **b**), ethanol production (**c** and **d**), glucose consumption (**e** and **f**), ethanol production yield (**g** and **h**), and ethanol production rate (**i** and **j**) were determined

We next monitored the ethanol fermentation performances of the multiple stress-tolerant C3253, C3751, and C4377 strains incubated in YPDA10 media (YPDA media containing 10% (w/v) glucose) at 30 and 40 °C up to 48 h. Although, at 30 °C, the growth rates of these multiple stress-tolerant strains was similar to that of the TISTR5606 strain (Fig. 6a), these strains showed significantly higher growth rates than the controls when incubated at 40 °C (Fig. 6b). At both 30 and 40 °C, most of the highest ethanol levels were obtained after 12-h incubation (Fig. 6c, d). Among these multiple stress-tolerant strains, the C3253 strain produced the highest ethanol levels at both 30 and 40 °C (31.37 and 30.08 g L^−1^ of ethanol, respectively) (Fig. 6c, d). While the ethanol production of C3751, C4377, and industrial TISTR5606 strains were at similar levels, which were higher than that of the W303 strain (Fig. 6c, d). In all cases, glucose consumption rates were relatively correlative with ethanol productivities (Fig. 6e, f). After fermentation for 12 h at 30 and 40 °C, the C3253 strain, which produced the highest levels of ethanol, exhibited the highest ethanol yields of 0.35 and 0.34 g ethanol/g glucose consumed and the highest ethanol production rates of 4.97 and 4.71 g L^−1^ h^−1^, respectively (Fig. 6g–j). Although, with fermentation performed at 30 °C, the ethanol yields and production rates of TISTR5606, C3751, and C4377 strains were similar, these two multiple stress-tolerant strains showed significantly higher ethanol yields and production rates than the TISTR5606 at high temperature (40 °C) (Fig. 6g–j). Based on our observations, the ethanol fermentation performances of these three multiple stress-tolerant strains were stable even at high temperature (at least up to 40 °C) and, among these, the C3253 strain showed the highest performance for ethanol fermentation at both normal and high temperatures, suggesting its high potential for use in high-temperature ethanol fermentation at industrial scale.

## Discussion

In this study, we found that our natural isolates of *S. cerevisiae*, which have been isolated from Thai fruits based on their thermotolerant characteristics (Auesukaree et al. 2012), were resistant to not only high temperature but also other environmental stresses including ethanol, osmotic, oxidative, and cell wall stresses. Consistent with our findings, the thermotolerant *S. cerevisiae* KNU5377 strain has been shown to be tolerant to ethanol, and oxidative stresses (Kim et al. 2013). In addition, several natural isolates of *S. cerevisiae* have been reported to be resistant to multiple stresses. For instance, the *S. cerevisiae* strains isolated from soil sample collected from a thermal power plant were thermotolerant and osmotolerant (Sree et al. 2000) and the wild isolates of *Saccharomyces* spp. yeasts were tolerant to multiple stresses present during lignocellulosic bioethanol fermentation (Wimalasena et al. 2014). Based on these observations, it is likely that natural yeast strains may have the capability to tolerate multiple stresses, possibly due to the fact that natural yeasts are constantly exposed to environmental fluctuations in their habitats.

During fermentation, yeast cells are continuously exposed to various stresses such as osmotic, ethanol, and oxidative stresses (Gibson et al. 2007). To cope with this multiple stress, cellular mechanisms responsible for protecting yeast cells from a wide range of physical and chemical stressors are indispensable. In this study, we found that, in response to heat, ethanol, osmotic, and oxidative stresses, the expression of *HSP* genes and the intracellular trehalose accumulation in the multiple stress-tolerant C3253, C3751, and C4377 strains were maintained at high levels, which were correlated with their growth rates under these stress conditions. Consistent with these findings, we have previously reported that the other two thermotolerant C3723 and C3867 strains also exhibited continuous expression of *HSP* and trehalose biosynthesis genes, i.e., *SSA4*, *HSP82*, *TPS1*, and *NTH1*, during exposure to long-term heat stress (Auesukaree et al. 2012). Based on our observations, it is likely that both HSPs and trehalose are important for an acquisition of tolerance against several environmental stresses, especially protein damage-inducing stresses. In agreement with our findings, the correlation between intracellular trehalose content and resistance to multiple stresses has been reported. For instance, the mutants unable to produce trehalose (i.e., the *Δtps1Δtps2* and *Δtps1Δhxk2* mutants) were more sensitive to osmotic stress than the wild-type strain (Hounsa et al. 1998). In addition, the levels of intracellular trehalose have been shown to be correlated with cellular resistances to high temperature, ethanol, and oxidants (Benaroudj et al. 2001; Mahmud et al. 2010). Interestingly, although the levels of intracellular trehalose in all strains tested were significantly increased in response to sorbitol-induced osmotic stress, the trehalose biosynthesis was not stimulated by high concentrations of glucose. Consistent with our result, the trehalose accumulation has been previously shown to start after glucose exhaustion (Lillie and Pringle 1980). Furthermore, the recent study on transcriptional regulatory network revealed that Tps2p and Tsl1p, the phosphatase and regulatory subunits of trehalose-6-phosphate synthase/phosphatase complex, respectively, are negatively controlled by the glucose signalling pathway in order to regulate the availability of storage carbohydrate (Apweiler et al. 2012). Based on this evidence, the trehalose biosynthesis seems to be inhibited by high glucose concentrations.

Our results also revealed the effects of high temperature, ethanol, high glucose, and H_2_O_2_ on inducing endogenous oxidative stress and cell wall stress. Consistent with our findings, the increased intracellular ROS level and the cell wall remodeling were observed after ethanol challenge (Du and Takagi 2007; Teixeira et al. 2009), and some components of the CWI pathway, such as Pkc1p, Bck1p, and Slt2p, were essential for cell survival under heat, ethanol, and oxidative conditions (Auesukaree et al. 2009; Fujita et al. 2006; Vilella et al. 2005). We found that the abilities to effectively induce cell wall remodeling and to maintain redox homeostasis are necessary for protecting yeast cells against several environmental stresses present during fermentation. It is possible that the ability to minimize the intracellular ROS levels in these multiple stress-tolerant strains may be due to high ROS-scavenging activities and/or effective stress responses. In agreement with this idea, it has been shown that, during fermentation, the thermotolerant KNU5377 strain exhibited a high expression of Yap1p, a major transcription factor involved in activating the transcription of antioxidant genes in response to oxidative stress (Kim et al. 2013), suggesting its high ability to cope with oxidative stress and to maintain redox balance. Previously, it has been shown that an increase of β-1,6-glucan levels was associated with a reduced sensitivity of yeast cells to Zymolyase, suggesting that the degree of β-1,6-glucosidic cross-linking between β-1,3-glucan, mannoprotein and chitin may contribute to cell wall robustness (Aguilar-Uscanga and Francois 2003). It is therefore possible that our multiple stress-tolerant strains may have high degrees of cross-linking between cell wall components, thereby leading to highly robust cell walls. Among the cellular responses tested, the cell wall remodeling and the maintenance of redox balance seem to be the primary defense mechanisms involved in the protection against cell damage induced by simultaneous multi-stress mimicking fermentation stress. If this is the case, the activation of other cellular responses towards cell damage, such as HSPs and trehalose, may be unnecessary. In agreement with our idea, the expression of genes involved in cell wall organization (such as *CSR2, KRE1, TSC11, GSC2*, *PIR3*, *SED1* and *SPI1*) and oxidative stress response (such as *GPX1, CTA1, SKN7,* and *SRX1*) were upregulated during sake brewing (Wu et al. 2006) and wine fermentation (Marks et al. 2008). Further studies are, however, required to clarify the precise molecular mechanisms underlying multiple stress tolerance of these strains.

In conclusion, we found that the C3253, C3751, and C4377 *S. cerevisiae* strains, which have been reported as thermotolerant yeasts, are resistant to multiple stresses, i.e. heat, ethanol, osmotic, and oxidative stresses. Our results revealed that continuous expression of *HSP* genes, intracellular trehalose accumulation, maintenance of redox balance, and effective cell wall remodeling are important for protection against various stresses. Nevertheless, cell wall remodeling and redox homeostasis seem to be major mechanisms required for tolerance to simultaneous multi-stress. Moreover, these strains displayed better performances of ethanol production than the control strains at both optimal and high temperatures.

## Abbreviations

CFW: calcofluor white
CWI: cell wall integrity
DCFH-DA: 2′:7′-dichlorodihydrofluorescein diacetate
HOG: high osmolarity glycerol
HSF: heat shock transcription factor
HSP: heat shock protein
PBS: phosphate-buffered saline
ROS: reactive oxygen species
TCA: trichloroacetic acid
